# Functional Conservation of *Asxl2*, a Murine Homolog for the Drosophila Enhancer of Trithorax and Polycomb Group Gene *Asx*

**DOI:** 10.1371/journal.pone.0004750

**Published:** 2009-03-09

**Authors:** Heather A. Baskind, Lucy Na, Quanhong Ma, Mayur P. Patel, David L. Geenen, Q. Tian Wang

**Affiliations:** 1 Department of Biological Sciences, University of Illinois at Chicago, Chicago, Illinois, United States of America; 2 Department of Medicine, Section of Cardiology and the Center for Cardiovascular Research, University of Illinois at Chicago, Chicago, Illinois, United States of America; Ecole Normale Supérieure de Lyon, France

## Abstract

**Background:**

Polycomb-group (PcG) and trithorax-group (trxG) proteins regulate histone methylation to establish repressive and active chromatin configurations at target loci, respectively. These chromatin configurations are passed on from mother to daughter cells, thereby causing heritable changes in gene expression. The activities of PcG and trxG proteins are regulated by a special class of proteins known as Enhancers of trithorax and Polycomb (ETP). The *Drosophila* gene *Additional sex combs* (*Asx*) encodes an ETP protein and mutations in *Asx* enhance both PcG and trxG mutant phenotypes. The mouse and human genomes each contain three *Asx* homologues, *Asx-like 1*, *2*, and *3*. In order to understand the functions of mammalian Asx-like (Asxl) proteins, we generated an *Asxl2* mutant mouse from a gene-trap ES cell line.

**Methodology/Principal Findings:**

We show that the *Asxl2* gene trap is expressed at high levels in specific tissues including the heart, the axial skeleton, the neocortex, the retina, spermatogonia and developing oocytes. The gene trap mutation is partially embryonic lethal and approximately half of homozygous animals die before birth. Homozygotes that survive embryogenesis are significantly smaller than controls and have a shortened life span. *Asxl2^−/−^* mice display both posterior transformations and anterior transformation in the axial skeleton, suggesting that the loss of *Asxl2* disrupts the activities of both PcG and trxG proteins. The PcG-associated histone modification, trimethylation of histone H3 lysine 27, is reduced in *Asxl2^−/−^* heart. Necropsy and histological analysis show that mutant mice have enlarged hearts and may have impaired heart function.

**Conclusions/Significance:**

Our results suggest that murine *Asxl2* has conserved ETP function and plays dual roles in the promotion of PcG and trxG activity. We have also revealed an unexpected role for *Asxl2* in the heart, suggesting that the PcG/trxG system may be involved in the regulation of cardiac function.

## Introduction

Polycomb Group (PcG) proteins and their antagonists, trithorax Group (trxG) proteins, were identified in *Drosophila* as transcriptional repressors and activators of homeotic genes (*Hox* genes), respectively [1]–[3]. Mutations in PcG and trxG genes disrupt the specification of anterior-posterior (A/P) positional information and lead to homeotic transformations. In addition to their roles in A/P patterning, PcG and trxG proteins are involved in many developmental processes and diseases [4]–[7]. They have been found to regulate the expression of hundreds of genes in mammals, insects, and plants.

PcG and trxG proteins function at the level of chromatin, and their functional mechanisms are highly conserved. PcG proteins function by forming three multi-protein complexes, PRC1, PRC2, and PhoRC. Genetic and biochemical studies have led to the current model in which the complexes work together to establish and maintain methylation marks, primarily on the tail of histone H3 [4]–[8]. The PhoRC complex contains sequence-specific DNA binding activity and also interacts with mono- and di-methylated lysine 27 of histone H3 (H3K27) [9]–[11]. It has been proposed that PhoRC plays the critical role of recognizing hypomethylated nucleosomes around upstream regulatory elements of PcG target genes. The PRC2 complex contains histone methyl-transferase (HMTase) activity that trimethylates H3K27 [12]–[14]. H3K27me3 is a well-known mark for silenced chromatin and is associated with promoters and regulatory elements of PcG target genes. The PRC1 complex binds H3K27me3 and prevents chromatin remodeling, thereby maintaining target chromatin regions in the silenced state [15], [16].

trxG proteins also function in multi-subunit complexes. Three trxG complexes, the SET1-like complex, the BRM complex and the MLL supercomplex, have been purified in mammalian cells. The SET1-like complex contains HMTase activities and trimethylates lysine 4 of histone H3 [17]. H3K4me3 is tightly associated with the promoter regions of transcriptionally active loci [18]–[19]. The BRM complex contains the SWI/SNF chromatin-remodeling ATPase BRM and mediates ATP-dependent nucleosome sliding [20]. The MLL supercomplex contains both HMTase activities and chromatin remodeling activities [21].

PcG and trxG mutations have opposite effects on axial patterning. PcG mutations cause posterior transformations and trxG mutations cause anterior transformations [22]. In addition, genetic experiments in Drosophila show that most PcG and trxG mutations are reciprocally suppressive. These observations are consistent with the opposing functions of PcG and trxG proteins to establish silenced and active chromatin structures, respectively. Surprisingly, mutations in a set of genes originally identified as PcG genes [*Asx*, *E(z)*, *E(Pc)*, *Psc*, *Scm and Su(z)2*] were later found to enhance trxG phenotypes [23]. Further studies suggest that these genes play dual roles in PcG-mediated transcriptional repression and trxG-mediated transcriptional activation of *Hox* genes. The genes were proposed to form the “Enhancers of trithorax and Polycomb” (ETP) group, which is distinct from both PcG and trxG [23]. More ETP genes have since been identified and added to the list [24]–[25]. Despite the importance of ETP genes in promoting PcG and trxG activity, the mechanism by which ETP proteins function is largely unknown. One hypothesis suggests that ETP proteins may help recruit PcG and trxG complexes to target chromatin. Consistent with this hypothesis, a few ETP proteins have been shown to associate with PcG complexes at least transiently [26]–[27]. Moreover, several ETP proteins have been localized to the nucleus and on polytene chromosomes, including two that bind to upstream maintenance elements of *Hox* genes [28]–[30].

The *Drosophila Additional sex combs* (*Asx*) gene was one of the 6 original ETP genes identified [23]. Mutations in *Asx* cause both posterior transformation (PcG phenotype) and anterior transformation (trxG phenotype) [31]. In addition, *Asx* genetically interact with both PcG and trxG genes [32]–[33]. Consistent with the hypothesized role of ETP proteins in PcG and trxG recruitment to target chromatin, Asx is a nuclear protein that binds distinct loci on polytene chromosomes [30]. However, it is not known with which PcG/trxG protein(s) Asx interacts, what biochemical activity it has, or which development process(es) it regulates. Most published studies on *Drosophila Asx* were performed with gain-of-function or hypomorph alleles, complicating the interpretation of data.

The mouse and human genomes each contain three *Asx* homologues, *Asx-like 1*, *2*, and *3* [34]–[36]. In order to understand the role of ETP in mammals, we have generated gene trap mutant mice for *Asx-like 2* (*Asxl2*). Analyses of *Asxl2* mutant mice suggest that murine *Asxl2* has conserved functions in the regulation of PcG and trxG. Furthermore, we found an unexpected role for *Asxl2* in the regulation of heart function.

## Results

### Generation of *Asxl2* mutant mice

**Figure 1:**
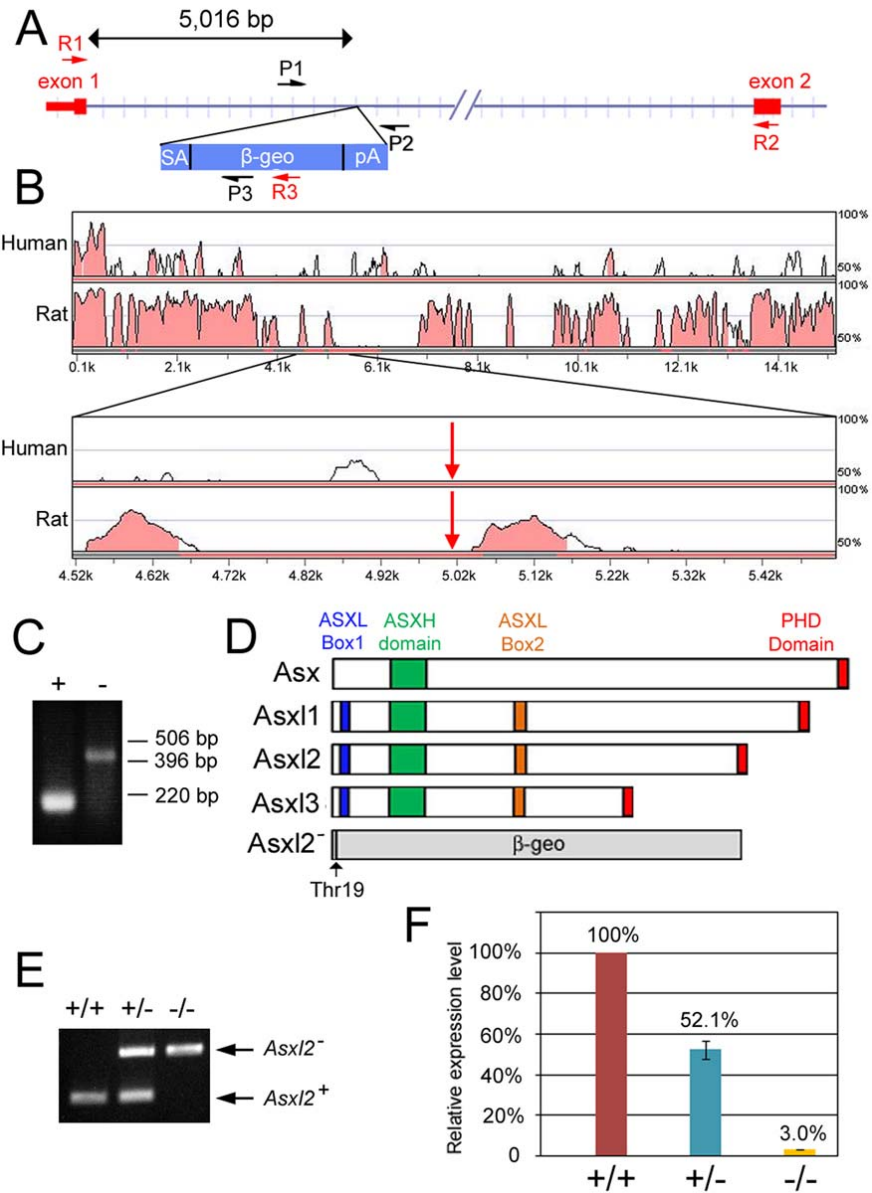
Generation of *Asxl2* mutant mice. (A) Schematic representation of the gene trap allele of murine *Asxl2* (not drawn to scale). Exon 1 contains the 5'UTR (the narrow portion of the red box) and coding sequence for the first 19 amino acids of Asxl2. The gene trap cassette (blue rectangle) is inserted 5,016 bp downstream of exon 1. P1, P2 and P3: primers used in genotyping PCRs. R1, R2 and R3: primers used in RT-PCR analysis of the wild-type and gene trapped transcripts. (B) Conservation between mouse *Asxl2* intron 1 and corresponding sequences in human *ASXL2* and rat *Asxl2* loci. The mouse, human and rat sequences were compared by SLAGAN. The 15,261-bp intron 1 of mouse *Asxl2* was used as the base sequence. The top two panels show distribution of conserved sequence modules within the entire length of mouse *Asxl2* intron 1. The bottom two panels show close-up views of 500 bp upstream and 500 bp downstream of the gene trap insertion site, which is indicated by red arrows. (C) RT-PCR analysis of the AQ0356 gene trap ES cell line. Transcripts from the wild-type (+) and mutant (−) alleles are detected with primer sets R1/R2 and R1/R3, respectively. The RT-PCR product in the mutant lane reflects splicing event that joins exon 1 with β-geo. (D) Schematic representation of the domain structures of Drosophila Asx, vertebrate Asxl1, Asxl2, Asxl3 and the predicted protein product from the *Asxl2^−^* allele. The ASXH (green box) and PHD (red box) domains are conserved from flies to mammals. The ASXL1 boxes 1 and 2 (blue and orange boxes) are conserved in the three mammalian Asx-like proteins but are not present in Asx. The mutant protein contains the first 19 amino acids of Asxl2 joined to β-geo. None of the conserved domains is present in the mutant protein. (E) PCR genotyping of genomic DNA from *Asxl2* wild-type (+/+), heterozygous (+/−) and mutant (−/−) mice. P1 and P2 generate a 250-bp product from the wild-type allele. P1 and P3 generate a 480-bp product from the mutant allele. (F) Real-time RT-PCR quantification of wild-type *Asxl2* transcripts in *Asxl2* wild-type (+/+), heterozygous (+/−) and mutant (−/−) hearts. The transcript levels in heterozygous and mutant hearts were 52.1% and 3.0% of that in wild-type hearts, respectively.

To generate mice mutant for *Asxl2*, we took advantage of the gene-trap ES cell line AQ0356 made by the Gene-Trap Consortium [37]. In the AQ0356 line, the gene trap cassette integrated within the first intron of *Asxl2*. We mapped the exact site of integration to 5016 bp downstream of exon 1 (Fig. 1A). Comparison of the sequence of the 15-kb *mAsxl2* intron 1 to homologous sequences in human and rat showed that the gene trap insertion does not disrupt any conserved sequence module (Fig. 1B). Using RT-PCR, we confirmed that transcription of the trapped allele (*Asxl2^−^*) produces an mRNA in which the first exon of *Asxl2* is joined to the gene trap cassette (Fig. 1C). The cassette contains a polyA signal at the 3′ end, interrupting endogenous splicing and causing translation to stop. Thus, the *Asxl2^−^* allele encodes a fusion protein containing the first 19 amino acids of Asxl2 joined to the β-geo reporter (Fig. 1D). This fusion protein is likely functionally null because it is missing all the conserved domains of wild-type Asxl2. We injected AQ0356 cells into mouse blastocysts to generate gene-trapped mice. The wild-type and the *Asxl2^−^* alleles are readily distinguishable by genotyping PCR (Fig. 1E).

To determine whether the insertion of the gene trap cassette prevents the production of wild-type *Asxl2* transcript, we have used real-time PCR to measure the level of *Asxl2* transcripts in the heart, where *Asxl2* is normally expressed abundantly. The level of *Asxl2* transcripts in the mutant heart is ∼3% of the wild-type level (Fig. 1F), suggesting that the gene trap insertion effectively though not completely prevented production of wild-type transcripts. The residual level of *Asxl2* transcripts could be the result of residual endogenous splicing, or it could represent a low level of transcription isoform produced from an alternative promoter downstream of the gene trap integration site. We conclude that *Asxl2^−^* is a severe loss-of-function allele.

### The expression pattern of the *Asxl2* gene trap in developing embryos and postnatal tissues

**Figure 2:**
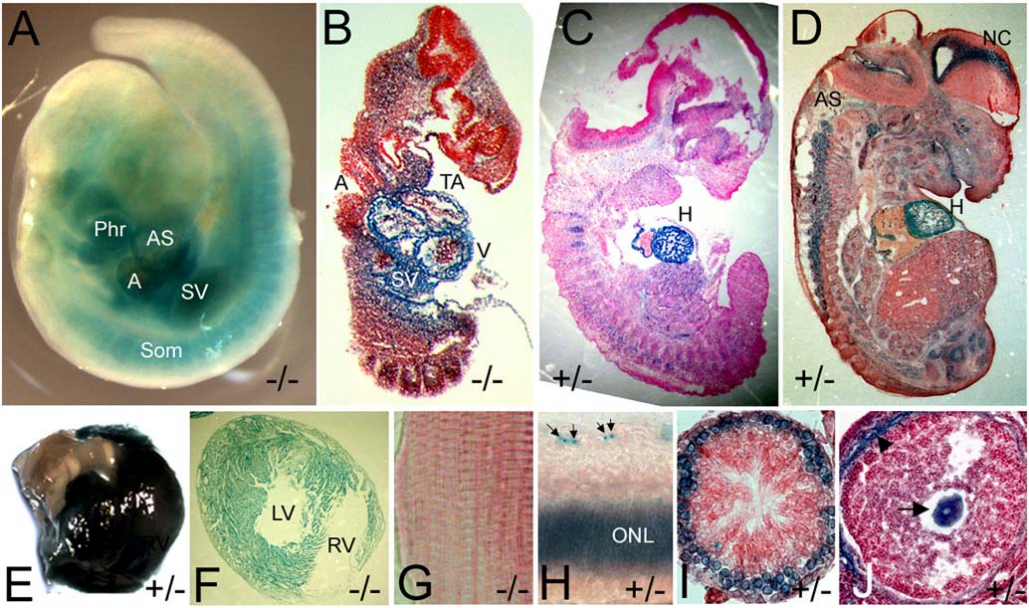
The expression pattern of the *Asxl2* gene trap in developing embryos and adult organs. Developing embryos and postnatal organs were stained with X-gal. (A) A whole-mount E9.5 embryo. (B–D) Cryo-sections of whole-mount stained E10.5 (B), E12.5 (C) and E14.5 (D) embryos. (E) Top view of a whole-mount stained postnatal heart showing strong X-gal staining in the heart but not in the aorta. (F) Cryo-section of a whole-mount stained postnatal heart. (G) Cryo-section of a piece of whole-mount stained skeletal muscle. The skeletal muscle in (G) and the heart in (F) were taken from the same animal and stained together. There was no detectable X-gal activity in the skeletal muscle. (H) Cryo-section of the retina in a whole-mount stained eye. Arrows point to stained cells in the ganglion cell layer. (I) Cross-section of a seminiferous tubule in a whole-mount stained testis showing expression in the primary spermatocytes. (J) Cross-section of an early tertiary follicle in a whole-mount stained ovary, showing expression in the oocyte (arrow). The blue color around the periphery of the follicle (arrowhead) was non-specific and also detected in wild-type samples. Phr: 1st pharyngeal arch; A: common atrial chamber; AS: aorta sac; SV: sinus venosus; Som: somites; V: common ventricular chamber; TA: truncus arteriosus; H: heart; AS: axial skeleton; NC: neocortex; RV: right ventricle; LV: left ventricle; ONL: outer nuclear layer.

Because gene trapping puts the beta-gal reporter under the transcriptional control of the endogenous gene's promoter, *lacZ* expression often reflects the expression pattern of the endogenous gene. X-gal stainings showed that the *Asxl2* gene trap is expressed at low levels broadly and at high levels in a tissue-specific manner in developing embryos (Fig. 2A–D). In particular, the *Asxl2* gene trap is highly expressed in the heart at all developmental stages examined, starting in E9.5 embryos (Fig. 2A) and continuing throughout embryogenesis (Fig. 2B–D). Strong expression was also detected in the axial skeleton, where PcG/trxG proteins have a well-known role in the regulation of anterior-posterior patterning. Other embryonic organs that express the *Asxl2* gene trap at high levels include the neocortex, the spinal cord and the eye.

The postnatal expression of the *Asxl2* gene trap is highly tissue-specific. Expression was detected in the heart, the retina, the ovary and the testis (Fig. 2E, F, H–J), but not in the liver, the brain, the spleen, the pancreas, or the lung (data not shown). While the *Asxl2* gene trap is ubiquitously expressed in the cardiac muscle, it is not expressed in the skeletal muscle (compare Fig. 2F and 2G). In the retina, the *Asxl2* gene trap is expressed in the outer nuclear layer and in a subset of cells in the ganglion cell layer (Fig. 2H). The *Asxl2* gene trap is also expressed during gametogenesis of both sexes. Its expression during spermatogenesis is restricted to the primary spermatocytes and is not detectable in secondary spermatocytes and other post-meiotic (haploid) cells (Fig. 2I). In the ovary, the *Asxl2* gene trap is expressed in the oocyte in follicles of all stages (Fig. 2J and data not shown).

### *Asxl2* is required for embryonic and postnatal development

When *Asxl2^+/−^* animals were backcrossed to wild-type mice, the genotypes of pups segregated at the expected 1∶1 ratio. However, among 220 pups born to matings between heterozygous parents, only 28 were homozygous mutants (*Asxl2^−/−^*), significantly less than the Mendelian ratio (Table 1). Among the 28, 23 survived to weaning and 5 died within 2–3 days of birth. The number of homozygous pups was less than half of what was expected, suggesting that more than half of *Asxl2^−/−^* mice died before birth. The exact time and cause of embryonic loss has not been determined.

**Table 1:** Recovered progeny of intercrosses between *Asxl2^+/−^* mice.

	Total number of pups	Genotype		
		+/+	+/−	−/−
Actual number	220	58	137	28
Expected* number	n/a	58	116	58
Chi square	n/a	n/a	0.935	10.465
*p* value	n/a	n/a	0.33	0.0012

* based on the number of wild-type pups.

**Figure 3:**
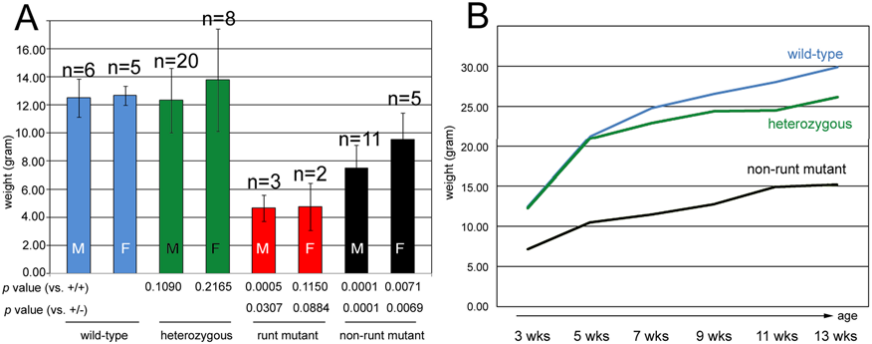
Growth defect in *Asxl2^−/−^* animals. (A) Comparison of body weights at weaning (3 weeks) for wild-type (blue columns), heterozygous (green columns), runt mutant (red columns) and non-runt mutant (black columns) mice. Error bars stand for standard deviation. M: males. F: females. (B) Growth of wild-type (blue line), heterozygous (green line) and non-runt mutant (black line) animals. Body weights were measured every two weeks after weaning and plotted against time. Animals of all three genotypes gained weight over time, but the weight gap between non-runt mutants and wild-type or heterozygous animals widens from 3 to 5 weeks and remains wide throughout the time course of measurements. The growth curve of runt mutants was not generated because very few runt mutants were born and all died early.

*Asxl2^−/−^* mice that survived past weaning fall into two categories. A small number (5 out of 23, or 21.7%) of the surviving mutants were runts. The runt mutants weighed an average of ∼4.7 grams at 3 weeks, less than 40% of the normal weaning weight (Fig. 3A). Although we kept the runt mutants with their mothers beyond the normal weaning age, the animals failed to thrive and remained extremely small. All runts died prematurely within two months of birth. The rest (18 out of 23, or 78.3%) of the mutants were also smaller than control littermates (Fig 3A), but they were successfully weaned and steadily gained weight as they grew older (Fig. 3B). We refer to these animals as non-runt mutants. The average weaning weights for non-runt mutant males and females were 7.5 grams and 9.5 grams, respectively (Fig 3A). Despite their small size, we did not find gross morphological abnormality in either runt or non-runt mutant mice. Because very few runt mutants were born and most of them died soon after birth, all the subsequent analyses were done with non-runt mutants.

### *Asxl2* mutant skeletons display both posterior and anterior transformations

**Figure 4:**
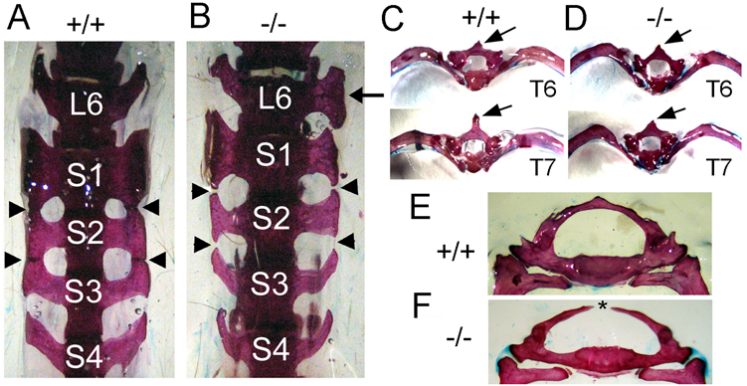
The loss of *Asxl2* disrupts axial skeleton patterning. (A, B) Ventral view of the sixth lumbar vertebra (L6) and the sacral vertebrae 1–4 (S1–4). (A) A wild-type skeleton. The wing-shaped lateral processes of S1, S2 and S3 fuse at the tip (arrowheads). The lateral processes of S4 do not fuse with those of S3. (B) A mutant skeleton. The lateral processes of S1, S2 and S3 fail to fuse. Arrowheads point to the sites at which fusion should happen. The lateral processes of mutant S2 resemble those of wild-type S3; those of mutant S3 resemble those of wild-type S4. One of the lateral processes of mutant L6 resembles that of S1 (arrow). (C, D) Caudal view of isolated T6 and T7 vertebrae, showing anterior transformation in the mutant. The wild-type T6 and T7 differ in the length of the neural spine (arrows) and the arch of the attached ribs (C). The mutant T7 resembles T6 in both the neural spine and the arch of ribs. (E, F) Caudal view of the first thoracic vertebrae (T1) in a heterozygous skeleton (E) and a mutant skeleton (F). The mutant T1 is split at the dorsal midline (asterisk).

PcG and trxG proteins play central roles in the repression and activation of *Hox* genes, respectively. In mouse PcG and trxG mutants, the expression of *Hox* genes is misregulated, leading to abnormal patterning of the axial skeleton. Consistent with the expression of the *Asxl2* gene trap in the axial skeleton (Fig. 2) and with the hypothesis that the mouse Asxl2 is part of the PcG/trxG/ETP system, analyses of whole-mount skeletons of *Asxl2^−/−^* mice revealed a number of axial skeletal abnormalities that are reminiscent of skeletal phenotypes observed in PcG, trxG, ETP and *Hox* mutant mice (Fig. 4, summarized in Table 2). The most penetrant phenotype involves the sacrum of all mutant mice. Mice normally have four sacral vertebrae (S1–4) with wing-shaped lateral processes. In all (7 out of 7) wild-type skeletons examined, S1–3 fuse at the tip of their lateral processes to form the articular surface that contacts the ilium, while S4 does not fuse with S3 (Fig. 4A). In contrast, the lateral processes of S1–3 fail to fuse in all (13 out of 13) *Asxl2^−/−^* skeletons examined (Fig. 4B). Absence of fusion between S1/S2 or S2/S3 suggests that S2 and S3 may have adopted a more posterior identity. This is supported by the observation that the lateral processes of mutant S3 resemble those of wild-type S4 (Fig. 4B) (100% penetrance). 46% (6 out of 13) of mutant skeletons also exhibit transformation of S2 to S3 (Fig. 4B, Table 2). A partial transformation of L6 to S1 was observed in a small number (2 out of 13) of mutant skeletons (Fig. 4B) but not in any wild-type or heterozygous skeleton. Because posterior transformation is a classical PcG mutant phenotype, these observations suggest that the loss of *Asxl2* disrupts PcG activity.

**Table 2:** Summary of skeletal defects observed in *Asxl2* deficient mice.

Genotype	+/+	+/−	−/−
#skeletons examined	7	10	13
Split C1	0	0	3 (23.1%)
Split C2	0	0	2 (15.4%)
Split C5	0	0	1 (7.7%)
Split C6	0	0	1 (7.7%)
Split T1	0	0	7 (53.8%)
T4 to T3	0	0	2 (15.4%)
T7 to T6	0	0	3 (23.1%)
Split T11	0	0	1 (7.7%)
T13 rib on one side	0	1 (10.0%)	0
L6 to S1 on one side	0	0	2 (15.4%)
S1/2 do not fuse	0	0	13 (100%)
S2 to S3^a^	0	0	6 (46.2%)
S2/3 do not fuse	0	5 (50.0%)	13 (100%)
S3 to S4^b^	0	0	13 (100%)

a The lack of S1/S2 fusion is not always accompanied by an obvious transformation of S2 to S3.

b The lack of S2/S3 fusion is not always accompanied by an obvious transformation of S3 to S4.

Anterior transformation was also observed in *Asxl2^−/−^* skeletons. In all wild-type skeletons examined, the 7^th^ thoracic vertebra (T7) and the 6^th^ thoracic vertebra (T6) can be distinguished by the length and shape of the neural spine and by the arch of the attached ribs (Fig. 4C). In 23% (3 out of 13) of mutant skeletons, there appeared to be two T6 vertebrae and no T7 vertebra because the mutant T7 resembles that of T6 in both neural spine and rib arch (Fig. 4D). Anterior transformation of T4 to T3 was also observed in 15% (2 out of 13) of mutant skeletons, but not in wild-type or heterozygous skeletons (Table 2). Because anterior transformation is a classical phenotype for trxG mutations, these observations suggest that the loss of *Asxl2* also disrupts trxG activity.

Another prominent feature of *Asxl2^−/−^*skeleton is splitting of the vertebrae at the dorsal midline, which was observed at multiple locations along the cervical and thoracic vertebral columns of *Asxl2^−/−^*animals (Fig. 4E, F and Table 1). Whether and how the split vertebrae are related to anterior or posterior transformation is unclear. However, split vertebrae have been previously observed in mice mutant for the ETP gene *Bmi-1*, for the PcG gene *Ring1a*, and for the gene *Taspase1* encoding an enzymatic processor of the trxG protein MLL [38]–[40]. Thus, the split vertebrae phenotype in *Asxl2^−/−^*skeleton is consistent with the hypothesis that murine Asxl2 functions in the same processes as PcG, trxG and ETP proteins do.

### *Asxl2* mutant animals die prematurely with enlarged hearts

**Figure 5:**
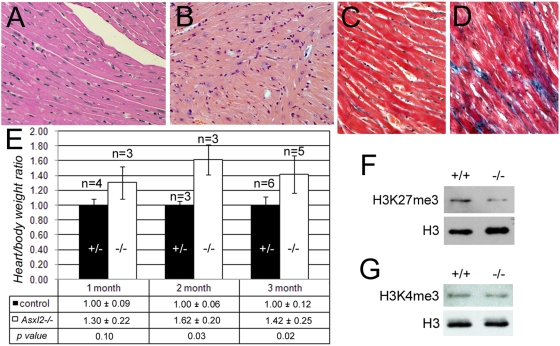
Cardiac phenotypes of *Asxl2^−/−^* animals. (A, B) H&E stainings of heart sections. The myocardial fibers, composed of cardiomyocytes, are well organized in the wild-type heart (A). In contrast, the heart of a moribund 5-month-old *Asxl2^−/−^* mouse shows cardiomyocyte disarray (B). (C, D) Examination of fibrosis in heart sections by Mason's trichrome (MTC) staining. Blue staining marks regions of fibrosis. The mutant (D) but not the wild-type (C) heart displays extensive interstitial fibrosis. (E) The heart/body weight ratios of mutant and control animals at 1 month, 2 months and 3 months after birth. The table below the chart shows the average ratio + standard deviation for each genotype and the p value for the time point. Error bars in the chart represent standard deviations. (F, G) Western blot analysis of the levels of H3K27me3 and H3K4me3 in wild-type and mutant hearts. As a control for equal loading, the blots were stripped and re-probed with an antibody against pan histone H3. The mutant heart has decreased level of H3K27me3 (F). There is no obvious change in the level of H3K4me3 (G).

Although the non-runt mutants lived past weaning and grew in size, they remained significantly smaller than control littermates. Furthermore, they became lethargic and moribund as they aged, dying prematurely between 2 and 7 months. Necropsy of three moribund non-runt mutant mice showed that they have disproportionally large hearts when compared to their small body size. Histological examination found signs of myocyte disarray and interstitial fibrosis in the hearts of older, moribund mutant animals (Fig. 5B, D), but not in age-matched control animals (Fig. 5A, C) or in younger mutants (data not shown). This suggests that the old mutants had decreased heart function and may have experienced heart failure. We compared the heart/body weight ratio of mutant animals at various ages with that of age- and sex-matched littermates. The average heart/body weight ratios were higher in mutant animals than in control animals at all three time points (Fig. 5E). The difference between mutants and controls were statistically significant in 2-month-old and 3-month-old animals (*p* = 0.03 and *p* = 0.02, respectively). This suggests that an enlarged heart could be a primary condition that existed before the animals became visibly sick. The increased heart/body weight ratio is unlikely an artifact of the mutant animals' growth defect, because the liver/body weight ratio is comparable between the mutant and control animals (data not shown). An enlarged heart in humans is a symptom associated with a number of cardiovascular diseases and is often found during heart failure. Although the exact cause of death is not known for *Asxl2^−/−^* animals, heart enlargement in these animals may have compromised heart function and contributed to the animals' deteriorating health.

### *Asxl2^−/−^* hearts have reduced level of H3K27me3

Histone methylation plays a central role in the mechanisms by which PcG and trxG proteins mark the chromatin of their target genes for repression or activation, respectively. The functions of PcG complexes converge on the trimethylation of lysine 27 of histone H3 [12]–[15], therefore the level of H3K27me3 provides a readout of overall PcG activity. The mechanisms by which trxG complexes modulate chromatin structure include both non-covalent, ATP-dependent nucleosome sliding and covalent modification of histone tails. Two of the three known mammalian trxG complexes contain HMTase activity that trimethylates lysine 4 of histone H3 (H3K4me3). Because the axial skeletal phenotypes of *Asxl2^−/−^* animals suggest that the loss of *Asxl2* disrupts PcG and trxG activities, we examined the global levels of H3K27me3 and H3K4me3 in the hearts of *Asxl2^+/+^* and *Asxl2^−/−^* animals. We found that mutant hearts had a lower level of H3K27me3 than wild-type hearts (Fig. 5F), suggesting that PcG-associated methyl-transferase activity is compromised in *Asxl2^−/−^* heart. The reduction in H3K27me3 levels in mutant hearts were observed in all four mutant-control littermate pairs that we examined. On the other hand, the level of H3K4me3 did not exhibit obvious difference between the same sets of mutant and wild-type hearts (Fig. 5G), suggesting that the HMTase activity of trxG complexes is not disrupted by the loss of *Asxl2*, or the disruption is subtle and hence difficult to detect by semi-quantitative Western blotting.

## Discussion

### Expression pattern of *Asxl2*

The *Asxl2* gene trap is expressed in a tissue-specific manner in both embryos and adult animals (Fig. 2). *Drosophila Asx* has been previously shown to be ubiquitously expressed from the blastoderm stage and on in the embryos [30]. There was no distinct tissue-specificity, though a relatively higher level of expression was found in the neurectoderm and later in the CNS. In developing mouse embryos, the *Asxl2* gene trap expression is also detected broadly at low levels. However, its expression is elevated in selected tissues, including in the heart and in the axial skeleton. The tissue-specific high level expression of the *Asxl2* gene trap is discernable in X-gal-stained whole-mount embryos (Fig. 2A) and is better resolved when the embryos are cryo-sectioned (Fig. 2B–D). In postnatal animals, expression of the *Asxl2* gene trap is highly tissue-specific (Fig. 2E–J). This differs from an earlier report on the analysis of *Asxl2* expression by Northern blot, which suggested broad and relatively uniform expression of *Asxl2* in six adult tissues including the kidney, the lung, the heart, the salivary gland, the skeletal muscle and the brain [34]. Highly similar Northern blot pattern was reported for *Asxl1* [34]. Although both gene trap and Northern blot detected *Asxl2* expression in the heart, the brain, and the eye, the expression of the gene trap is much more tissue-specific than what could be expected from the Northern blot. Notably, the gene trap is not expressed in the kidney, the lung, the skeletal muscle, or the postnatal brain.

One possible explanation for the discrepancy between our result and the previous reported Northern blot is that insertion of the gene trap disrupted regulatory element(s) within the first intron of *Asxl2*. Sequence comparison between mouse, human and rat *Asxl2* did not find conserved sequence modules at the site of gene trap integration (Fig. 1B). However, we cannot exclude the formal possibility that certain mouse-specific regulatory element(s) is disrupted by the gene trap or that the gene trap insertion disrupted critical spacing between regulatory elements. Alternatively, the Northern blot probe used for *Asxl2* may have cross-hybridized with *Asxl1* and/or *Asxl3* transcripts. The 1.1-kb probe was made from the EST clone AW496276, for which a partial sequence (588 bp) is available from the IMAGE consortium. We blasted the mouse genome with this partial sequence and found a 344-bp region and a 379-bp region that exhibit sequence homology (69% and above) with *Asxl1* and *Asxl2* transcripts, respectively. The entire 1.1-kb probe may contain additional homologous sequences. Finally, *Asxl2* gene has been suggested to have multiple transcript isoforms. If any of the isoforms is transcribed from an alternative promoter downstream of the gene trap cassette insertion site, it would not be detected in the X-gal stainings. Further analyses, including Northern blots and *in situ* hybridizations using highly *Asxl2*-specific probes and immunohistochemistry studies of the Asxl2 protein, are needed to determine the expression pattern of endogenous *Asxl2* and to fully understand the discrepancy between our result and previous report.

Interesting heart phenotypes and axial skeleton phenotypes were observed in *Asxl2* mutant animals, suggesting that the expression of the gene trap in the heart and in the axial skeleton is probably reflective of endogenous expression and function in these tissues. Because of the strong expression of the *Asxl2* gene trap in the heart and in the axial skeleton, the current report characterized the heart and skeleton phenotypes in further details and focused on the heart when examining the effects of the gene trap on endogenous *Asxl2* transcription (Fig. 1F) and on histone modifications (Fig. 5F, G).

The *Asxl2* gene trap is expressed in the retina (Fig 2H); Northern blot and EST profile also suggested that *Asxl2* is expressed in the eye [34]. Consistent with this expression, some *Asxl2* mutant mice have smaller, degenerating eyes compared to wild-type animals (data not shown). A role for *Asxl2* in the eye has been previously speculated because a balanced reciprocal translocation between human ASXL2 and the Gt4-2 locus was discovered in a patient with bilateral colobomas in the retina and iris [41]. Eye phenotypes in mice are severely influenced by genetic background. Due to the mixed genetic background of animals examined in the current report, the eye phenotypes of *Asxl2* mutant mice were not characterized.

### Functional conservation of *Asxl2*

Analyses of *Asxl2^−/−^* mice show that *Asxl2* has an important role in animal survival and growth. The loss of *Asxl2* activity results in partial embryonic lethality, shortened life span of adult animals, and a reduction in size and body weight. Similar phenotypes have been previously described for mice mutant for the PcG gene *M33* or the ETP gene *Bmi-1* [42]–[43]. Both M33 and Bmi-1 are associated with the PcG complex PRC1 [44]. Detailed analyses of the genetic and biochemical interactions between *Asxl2*, *M33* and *Bmi-1* are needed to shed light on whether and where Asxl2 regulates the function of the PRC1 complex.

*Asxl2^−/−^* mice exhibit a number of axial skeletal abnormalities (Fig. 4H), indicating that *Asxl2* has a role in the patterning of the axial skeleton, where PcG, trxG and other known ETP genes function. The trademark characteristic for ETP genes is their dual roles in promoting both PcG and trxG activities. Such dual roles have been shown for Drosophila *Asx*. We observed both posterior transformations (S3->S4 and S2->S3) and anterior transformations (T7->T6 and T4->T3) in *Asxl2^−/−^* skeletons, suggesting that the loss of *Asxl2* disrupts both PcG and trxG activities in the axial skeleton (Fig. 4). A compromise in PcG-associated HMTase activity was also observed in *Asxl2^−/−^* hearts, which exhibit reduced global level of H3K27me3 (Fig. 5F). It has been previously shown that knockdown of E(z), the HMTase subunit of PRC2, abolishes H3K27me3 in Drosophila [45]. Similarly, knocking down of EZH2, a human homolog of E(z), led to a global decrease in H3K27me3 in cancer cells [46]. We notice that while H3K27me3 is completely abolished in E(z) knockdown cells, it is significantly reduced but not abolished in EZH2 knockdown cells or in *Asxl2^−/−^* hearts. A likely explanation is functional redundancy between multiple homologs in mammals. Ezh1, the other mammalian homolog for E(z), may be partially redundant with Ezh2. Similarly, Asxl1 and Asxl3 may be partially redundant with Asxl2. Alternatively, *Asxl2^−/−^* hearts may have residual Asxl2 activity since the gene trap does not completely abolish wild-type *Asxl2* transcripts (Fig. 1F).

In contrast to H3K27me3, the level of H3K4me3 did not exhibit obvious change in *Asxl2^−/−^* hearts (Fig. 5G). This observation could suggest a tissue difference in Asxl2's function: it may regulate trxG activity in the axial skeleton but not in the heart. However, there are several alternative explanations. It is possible that Asxl2 regulates trxG activity at only a subset of trxG target loci. In this case, the loss of *Asxl2* may reduce H3K4me3 at the promoters of these loci without significant impact on the level of bulk H3K4me3. Testing this hypothesis will require the identification of Asxl2 target loci and he examination of local H3K4me3 levels by chromatin immunoprecipitation (ChIP). Alternatively, Asxl2 may specifically regulate the ATP-dependent chromatin remodeling activity but not the HMTase activity of trxG complexes. It will be interesting to compare the chromatin remodeling activity of trxG complexes purified from wild-type and *Asxl2^−/−^* hearts in the future. A third possibility is that there may be a division of labor among mammalian Asx-like proteins, so that Asxl2 primarily regulates PcG activity while trxG activity is primarily regulated by Asxl1 or Asxl3. This last scenario would predict that *Asxl2* is more essential for PcG activity than for trxG activity. Consistent with this scenario, even though both posterior transformations and anterior transformations were observed in *Asxl2^−/−^* skeletons, posterior transformations have a higher penetrance (100% for S3->S4 transformation vs. 23% for T7->T6 transformation). The generation of *Asxl1* and *Asxl3* mutant animals will allow us to address the potential division of labor among Asx-like proteins.

### Role of *Asxl2* in the heart

A PcG gene, *Rae28*, has previously been shown to be required for sustaining the expression of the cardiac selector gene *Nkx2.5* in developing mouse embryos [47]. The loss of *Rae28* disrupts an early and important step of cardiac morphogenesis, cardiac looping, that takes place between E8.5 and E9.5. *Asxl2^−/−^* animals that survived embryogenesis do not display any obvious abnormalities in the gross morphology of the heart (data not shown). However, some *Asxl2^−/−^* embryos died *in utero* and could have more severe phenotypes than the ones that were born. A detailed phenotypic characterization of *Asxl2^−/−^* embryos is needed to answer whether Asxl2 plays a role in heart morphogenesis. The variability in survival and/or in the severity of phenotypes between individual embryos could be due to variation in residual Asxl2 activity or functional redundancy among Asx-like proteins.

In postnatal animals, cardiac function is known to be epigenetically regulated. For example, class I and class II histone deacetylases (HDACs) promote and inhibit cardiac hypertrophy, respectively [48]–[49]. However, whether the PcG/trxG system plays a role in postnatal heart is unclear, partly because the embryonic lethality of many PcG/trxG mutations precluded functional study of postnatal development. The *Asxl2^−/−^* animals provide a good opportunity to address this question. A molecular signature of cardiac hypertrophy is the reactivation of multiple genes that are active during fetal development but repressed in the adult heart [50]. Given the role of PcG proteins in the maintenance of silenced loci, PcG proteins could be involved in the repression of these genes in normal hearts. Two important research directions in the future will be the full characterization of the heart phenotypes of *Asxl2^−/−^* animals and the study of the underlying mechanisms, especially the role of the PcG/trxG system.

## Materials and Methods

### Generation of *Asxl2* mutant mice

The AQ0356 gene trap ES cell line was purchased from the Sanger Institute. The gene trap event in AQ0356 ES cell line was confirmed with RT-PCR and with sequencing. For RT-PCR, total RNA was extracted with Trizol (Invitrogen). 1 µg of RNA was reverse transcribed with the R3 primer and SuperScript II reverse transcriptase (Invitrogen). Reverse transcription using the R2 primer was performed in a separate tube as a control. PCR reactions were performed using 1/7 of the cDNA product and AmpliTag Gold (ABI). The anneal temperature was 58°C and 30 cycles of reactions were performed. Sequences for RT-PCR primers are:

R1: 5′-GCCTCCCAGTCAGTCAGAAC-3′;
R2: 5′-GTCCTTCTCTCTGGATAACC-3′;
R3: 5′-GTAATGGGATAGGTCACGT-3′.

The RT-PCR product of the mutant allele was sequenced to confirm that the beta-geo reporter is joined to exon 1 after splicing. AQ0356 ES cells were then expanded and injected into blastocysts. Microinjection was performed by the Stanford Transgenic Facility. Chimera mice were mated and the progeny were screened by genotyping PCR for germ-line transmission. Three independent lines, derived from three chimeras, were bred. The three lines were indistinguishable in gene trap expression pattern and in phenotypes. All the animals analyzed were in C57BL/6J-129S2 mixed genetic background. The animal protocols were approved by the Animal Care Committee (ACC) at the University of Illinois at Chicago.

### Mapping gene trap integration site

A set of 30 forward primers that scan the 15-kb intron 1 of *Asxl2* at 0.5-kb interval were designed. PCR reactions were performed using individual forward primer and the P3 reverse primer. An extension time of 1 min. was used in the PCR reactions. Primers #10 and #11 generated PCR products. The two products differ in length by ∼0.5 kb, correlating with the ∼0.5 kb distance between the two primers. The PCR products were then sequenced to determine where genomic sequence is joined to gene trap cassette sequence.

### Genotyping PCR

Genomic DNA was prepared from clipped mouse tails following standard protocols. PCR reactions were performed with the PCR Master Mix (Qiagen). Sequences for genotyping PCR primers are:

P1: 5′-CCAACCCAACAGTCAGTATG-3′,
P2: 5′-CAAATCTGCCTCATCCTCTC-3′,
P3: 5′-CTCTCCCATCCACTACTCAG-3′

### Real-time RT-PCR

Total heart RNA was extracted with Trizol (Invitrogen) from two sets of wild-type, heterozygous and mutant mice. The RNA was treated with DNase I (Fermentas) and purified with RNeasy columns (Qiagen). Real-time RT-PCRs were performed on a DNA Engine Opticon 2 system (Bio-Rad) using the SuperScript™ III Platinum SYBR Green One-Step qRT-PCR kit (Invitrogen). Three primer pairs were tested. A pair of primers that straddle exons 9 and 10 of *Asxl2* exhibit the best melting curve and were used to generate the data. 300 ng total RNA was used as template in each reaction. Reaction conditions: 50°C for 15 min, 95°C for 5 min, and 40 cycles of 95°C for 15 s, 60°C for 30 s, and 70°C for 1 min. The level of *Asxl2* transcripts was normalized against that of *β-actin* transcripts in the same sample. Triplicate reactions were performed for each primer pair (*Asxl2* or *β-actin*) and each set of RNA samples.

Sequences of *Asxl2* primers are:

Forward: 5′-CTCCTGAAATGCAGGTGAGA-3′ (within exon 9)
Reverse: 5′-TTGCTTTGGGATCACTTGAG-3′ (within exon 10)

Sequences of *β*-actin primers are:

Forward: 5′-TCACCCACACTGGCCCATCTACGA-3′
Reverse: 5′-TGGTGAAGCTGTAGCCACGCT-3′

### X-gal staining of embryos and postnatal tissues

Postnatal tissues and embryos were fixed at 4°C in 4% paraformaldehyde (PFA) for 1–2 hours. After fixation, postnatal tissues were cross-sectioned or cut longitudinally into halves and washed with Tissue Rinse A (2 mM MgCl_2_, 5 mM EGTA in PBS) followed by Tissue Rinse B (2 mM MgCl_2_, 0.01% w/v sodium deoxycholate, 0.02% w/v NP-40 in PBS) for 30 minutes each at room temperature. Embryos were rinsed three times in Tissue Rinse B for 10 minutes each. All specimens were transferred to Staining Solution (5 mM potassium ferrocyanide, 5 mM potassium ferrocyanide, 1 mg/ml X-gal in Tissue Rinse B) and stained at 37°C for 5 hours per day and stored at 4°C until color developed. Tissues and embryos were then post-fixed in 4% PFA, equilibrated in 30% sucrose and embedded in OCT.

### Staining of axial skeletons

Adult mice carcasses were skinned and eviscerated. The skeletons were fixed in 95% ethanol for 4 days and defatted in acetone for 2 days. The skeletons were then incubated at 37°C in a staining solution of 0.005% alizarin red (Sigma A5533), 0.015% alcian blue (Sigma A3157), 90% ethanol, and 5% glacial acetic acid for 3 days. The skeletons were rinsed in distilled water and cleared in 1% KOH for 2 days. They were then switched into 0.8% KOH and 20% glycerol for 2 weeks, 0.5% KOH and 50% glycerol for 1 week, 0.2% KOH and 80% glycerol for 1 week, and finally stored in 100% glycerol.

### Histology

Mice were euthanized by CO_2_ inhalation followed by cervical dislocation. Hearts were immediately harvested, perfused with 10% formalin, and fixed in 10% formalin. Paraffin-embedding and sectioning were performed at the Veterinary Diagnostic Laboratory, University of Illinois at Urbana-Champaign. H&E stainings were performed following standard protocols. For Mason's trichrome staining, 10 µm paraffin-embedded sections were rehydrated and then incubated in pre-warmed Bouin's Solution in a 60°C water bath for 1 hour. After rinsing in running tap water, the slides were incubated in Working Weigert's Hematoxylin solution (Sigma HT1079) for 5–15 minutes, depending on the age of the solution. The slides were rinsed in running tap water and then stained in Biebrich Scarlet-Acid Fuschin solution (Sigma HT151) for 5 minutes. The slides were rinsed in running tap water and placed in 25% Phosphomolybdic acid solution/25% Phophotungstic acid solution (Sigma HT153, HT152) for 10 minutes. The slides were placed in Aniline Blue solution (Sigma HT154) for 5 minutes, rinsed in distilled H_2_O and placed in 1% acetic acid for 1 minute. Finally, the slides were dehydrated through a series of alcohols to xylene and mounted using Cytoseal XYL Mounting Medium (Thermo Scientific DXD-10140747).

### Western blotting

Mice were euthanized by CO_2_ inhalation followed by cervical dislocation. Hearts were immediately harvested and flash frozen in a dry ice-ethanol bath. Frozen hearts were cut to small pieces on dry ice and homogenized in hypotonic buffer (PBS containing 0.5% Triton X 100) supplemented with protease inhibitors cocktail in a dounce homogenizer. Nuclei were collected by centrifugation at 6,000 g for 15 minutes at 4°C and washed once with hypotonic buffer. Histones were extracted over night at 4°C from the nuclei pellet by 0.2N HCl. Insoluble fraction was cleared by centrifugation at 6,000 g for 15 minutes at 4°C. The supernatant (histone extract) was flash frozen in a dry ice-ethanol bath after the protein concentration was measured by BCA assay. Total histones was separated on 15% SDS-PAGE gels. After transfer, membranes were blotted with mouse anti-H3K27me3 or rabbit anti-H3K4me3. As a loading control, blotted membranes were stripped, the completeness of stripping was confirmed by ECL reaction, and stripped membranes were blotted with rabbit anti-pan histone H3. All antibodies were purchased from Active Motifs.

### Statistical analyses

Paired *t*-tests and Chi-square tests were performed using GraphPad Software on-line statistical calculators (http://www.graphpad.com/quickcalcs/ttest1.cfm and http://www.graphpad.com/quickcalcs/chisquared1.cfm). Paired *t*-tests were used to determine whether the weight difference between mutants and controls were statistically significant (Figure 3A) and whether the difference in the heart/body weight ratios of mutant and control animals was statistically significance (Figure 5E). Two-tailed *p* values were calculated and shown in the figures. Chi-square tests were used to determine whether the actual numbers of heterozygous and mutant animals born were statistically different from those expected based on the number of wild-type animals. Two-tailed *p* value was calculated for each genotype.
